# Environmental Filtering of Bacterial Communities Driven by Pesticide Residue Profiles in the Almaty Region, Kazakhstan

**DOI:** 10.3390/biology15090712

**Published:** 2026-04-30

**Authors:** Lazzat Asylbekkyzy, Bekzhan D. Kossalbayev, Fiaz Ahmad, Jingjing Wang, Assemgul K. Sadvakasova, Meruyert O. Bauenova, Altynbek A. Abseyt, Dilnaz E. Zaletova

**Affiliations:** 1 Department of Biotechnology, Faculty of Biology and Biotechnology, Al-Farabi Kazakh National University, Al-Farabi 71, Almaty 050038, Kazakhstan; lazzat.asylbekkyzy01@gmail.com (L.A.); bauyen.meruyert@gmail.com (M.O.B.);; 2 Department of Chemical and Biological Technologies, Satbayev University, 22 Satpaev Street, Almaty 050013, Kazakhstan; 3 Ecology Research Institute, Khoja Akhmet Yassawi International Kazakh-Turkish University, Turkistan 161200, Kazakhstan; 4 Key Laboratory for Space Bioscience & Biotechnology, School of Life Science and Technology, Northwestern Polytechnical University, Chang’an Campus, Xi’an 710072, China; 5 Tianjin Institute of Industrial Biotechnology, Chinese Academy of Sciences, No. 32, West 7th Road, Tianjin Airport Economic Area, Tianjin 300308, China

**Keywords:** soil microbiome, pesticide residues, r/K-selection strategies, 16S rRNA sequencing, bioremediation

## Abstract

Soil contamination by complex pesticide mixtures poses a systemic threat to ecosystem health, yet the mechanisms of microbial community assembly under the coexistence of legacy and modern pollutants remain insufficiently understood. This study evaluated the influence of legacy organochlorine pesticides (OCPs) versus current-use agrochemicals on the structure and inferred functional potential of soil bacterial communities in the Almaty Region, Kazakhstan, using high-throughput 16S rRNA gene sequencing and GC–MS/MS analysis of 217 compounds. Results revealed a clear contrast between contamination regimes: modern organophosphate insecticides and herbicides, such as simazine (up to 32.3 mg kg^−1^ at the Amangeldy site), were associated with lower alpha diversity (Shannon ≈ 3.03) and enrichment of copiotrophic taxa such as *Pseudomonas* and *Sphingobium*. In contrast, persistent OCP residues, such as p,p′-DDE (up to 1.43 mg kg^−1^ at the Kyzylkairat site), were associated with higher diversity (Shannon ≈ 5.46) and enrichment of more stress-tolerant oligotrophic lineages, including *Acidobacteria* and Vicinamibacteraceae. Procrustes analysis supported significant concordance between pesticide profiles and taxonomic structure (M^2^ = 0.286, *p* < 0.001), indicating that pesticide residue composition was strongly associated with bacterial community structure across the studied soils. The observed shift in community balance, particularly the relative increase in *Pseudomonas* versus *Acidobacteria*, is proposed as a candidate compositional indicator of ecosystem instability in semi-arid agricultural soils and may inform future remediation-oriented studies.

## Simple Summary

Farmers in southern Kazakhstan often face low crop yields because many soils are salty, dry, and poor in nutrients, and standard fertilizer use is often inefficient under these conditions. This study explores whether a new sowing approach can help cotton and maize grow better on degraded soils in the arid Turkestan region. The approach combines a water-holding hydrogel mixture, mineral fertilizers, beneficial microorganisms, and a machine that places these materials close to the seed in a single field operation. This study compares two placement methods to determine which one better supports early plant growth, improves soil moisture, increases nutrient availability, and raises crop productivity while reducing losses of water and fertilizers. The results are expected to support the development of a practical technology for restoring low-fertility soils and improving the stability of crop production in dry areas. This is important for society because it may help farmers use scarce water and inputs more efficiently, reduce pressure on degraded land, and strengthen food production in regions affected by drought and salinity.

## 1. Introduction

Intensive anthropogenic pollution, driven by the excessive global application of agricultural pesticides, poses a systemic threat to ecosystem integrity and public health [1,2,3]. Although these chemical agents are designed to control agricultural pests, their high environmental persistence and capacity for bioaccumulation in soil and water networks induce severe toxicological, neurotoxic, and endocrine-disrupting effects in humans and livestock [4,5,6]. To mitigate these global risks, the Stockholm Convention was established to regulate the management of persistent organic pollutants (POPs) [7]. Nevertheless, many parent compounds can transform within the soil matrix into highly recalcitrant and toxic metabolites that actively enter trophic chains. Within this context, priority pesticides and their degradation products, specifically organochlorine compounds (DDT and hexachlorocyclohexane isomers, including lindane) and organophosphate insecticides (phosalone, parathion), along with triazine herbicides (simazine) and fungicides (triadimefon), are of particular concern as they form highly toxic, complex chemical cocktails in soils. The survival and adaptation of microorganisms in these extreme anthropogenic niches require specific metabolic and physiological modulations essential for environmental safety and ecosystem stability [8].

In Kazakhstan, the challenge of pesticide contamination is exacerbated by a complex intersection of contemporary agrochemical practices and a massive toxic legacy from the former Soviet Union [9]. Between 1960 and 1990, OCPs were applied massively across Central Asian agricultural landscapes to protect cereal crops, vegetables, and orchards. Although their usage was officially restricted in the 1970s, enormous quantities of obsolete and unusable chemicals remain stockpiled across the republic [10]. The Almaty Region represents a critical zone of ecological concern, harboring 64 abandoned storehouses containing approximately 68.4 tons of degraded agrochemicals [11]. The physical deterioration of these facilities has led to the continuous leaching of contaminants into surrounding soils and groundwater, creating chronic environmental hazards in the vicinities of rural settlements such as Amangeldy and Kyzylkairat [12,13].

The ecological dynamics of these priority pollutants and their specific impacts on soil microbiome assembly are actively investigated globally using high-throughput 16S rRNA profiling, providing critical analogs to our research. International studies confirm that distinct chemical profiles act as strict deterministic filters, driving specific bacterial community trajectories. For instance, regarding historical organochlorine contamination, Sangwan et al. [14] investigated hexachlorocyclohexane (HCH)-polluted agricultural soils in India, demonstrating that chronic HCH exposure drastically reduces alpha diversity while strictly selecting for *Sphingobium* and *Pseudomonas* lineages. Similarly, Fang et al. [15] evaluated historical DDT contamination in Chinese agricultural soils, confirming that legacy DDT residues selectively favor stress-tolerant Actinobacteria and specific Proteobacteria, permanently altering the native community structure.

The impact of complex modern pesticide cocktails, particularly triazine herbicides, has been extensively documented; for example, simazine and its analogs profoundly restructure the rhizosphere microbiome by suppressing plant-beneficial symbionts while stimulating highly specific degradation networks [16].

This structural shift is accompanied by deep molecular adaptations, where organochlorine stress deterministically enriches virus-encoded metabolism and pesticide degradation-associated auxiliary genes within the soil microbiome [17]. Beyond localized taxonomic shifts, such chemical pressure has been shown to uniformly reshape beneficial microbial guilds across diverse global soil types, underscoring the predictable and deterministic nature of bacterial assembly patterns under chronic pesticide stress [18].

Soil microorganisms are not merely passive survivors under chemical stress; they can contribute to the transformation and natural attenuation of xenobiotics in contaminated environments. Crucially, the dominant taxa identified through our correlation analysis, most notably *Pseudomonas* and *Sphingobium*, align with lineages previously reported in pesticide-impacted soils. This pattern suggests that the observed compositional shifts are consistent with the enrichment of pesticide-associated taxa, although they do not by themselves distinguish degradation potential from stress tolerance. Accordingly, the increased relative abundance of these correlated taxa supports their potential utility as candidate compositional indicators of ecosystem disturbance under chronic xenobiotic pressure [19,20]. To address this critical knowledge gap, our study employs high-throughput 16S rRNA gene sequencing coupled with comprehensive chemical profiling (GC-MS/MS) to elucidate how microbiomes adapt to the chronic exposure of diverse xenobiotic classes. Ultimately, the primary objective of this research is to comprehensively investigate the ecological interactions between bacterial communities and pesticide-contaminated soils across the agricultural lands adjacent to the Amangeldy and Kyzylkairat settlements in the Almaty Region.

## 2. Materials and Methods

### 2.1. Study Area and Soil Sampling

**Figure 1:**
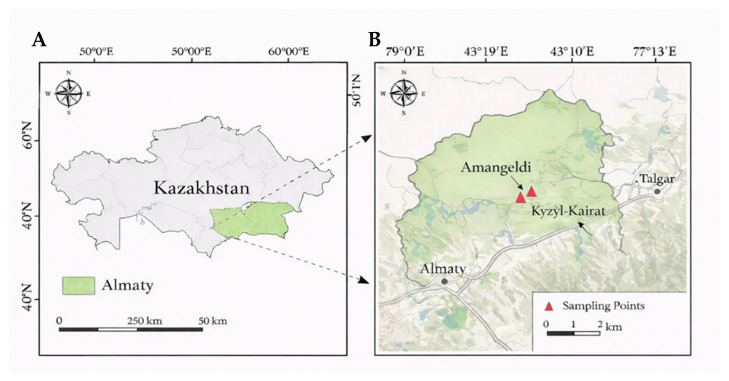
Study area and sampling site locations in the Almaty Region, Kazakhstan. Note: (**A**) Location of the Almaty Region within Kazakhstan. Note: (**B**) Detailed map showing the sampling sites (Amangeldy and Kyzylkairat).

Soil samples were collected in May 2025 from two settlements in the Almaty Region (Kazakhstan) characterized by long-term pesticide pressure: Kyzylkairat (43°17′58.8″ N, 77°11′40.3″ E), where a storage facility for previously widely used organophosphate pesticides is located, and Amangeldy (43°17′55.3″ N, 77°12′27.8″ E) (Figure 1), where the history of pesticide use is comparable and elevated concentrations of organochlorine and organophosphate compounds in soil have been reported [9,10]. To assess the spatial distribution of contamination, surface soil samples (approximately 5–10 cm depth) were collected at three distinct spatial zones relative to the pesticide warehouses at both sites. Specifically, the sampling points included: (1) inside the pesticide warehouse area (KIN and AIN), (2) in the immediate neighborhood adjacent to the warehouse (KNH and ANH), and (3) at a distance of 10 m from the warehouse (KD10 and AD10). A detailed description of the sampling locations, group codes, and corresponding coordinates is provided in Table 1.

To account for small-scale spatial heterogeneity, five independent topsoil cores were collected within a 10 × 10 m grid at each sampling zone using a sterile stainless-steel auger. These samples were treated as biological replicates for downstream microbiome analyses (*n* = 5 per zone). After collection, all samples were transferred into sterile glass containers, transported at 4 °C, and stored until processing. Before downstream analyses, soils were sieved through a 2 mm mesh to remove coarse debris and plant residues. Each processed sample was then divided into two fractions: one fraction was used for pesticide residue analysis by GC–MS/MS (Agilent Technologies, Santa Clara, CA, USA), and the other for microbiological analysis. All fractions were stored at −20 °C until further processing.

### 2.2. Pesticide Residue Analysis

To quantify baseline pesticide contamination, soil samples from all six sampling groups were subjected to chemical analysis. These groups represented two study locations, Kyzylkairat (KIN, KNH, and KD10) and Amangeldy (AIN, ANH, and AD10), sampled across three spatial zones relative to the pesticide warehouse at each site.

Pesticide concentrations were determined at Standard Sci-Tech Innovation (Qingdao, China) using a modified QuEChERS-based extraction procedure adapted for soil matrices. Briefly, approximately 5 g of homogenized soil was extracted with acetonitrile containing acetic acid. Extract cleanup was performed using MgSO_4_, PSA, and C18 sorbents. The cleaned extracts were evaporated under a nitrogen stream at 40 °C, reconstituted in ethyl acetate, and filtered through 0.22 μm membrane syringe filters prior to instrumental analysis.

Quantification was performed by GC–MS/MS on an Agilent 6890N system coupled to an Agilent 5975B mass spectrometer (Agilent Technologies, Santa Clara, CA, USA) and equipped with an HP-5MS capillary column (30 m × 0.25 mm × 0.25 μm; Agilent Technologies, Santa Clara, CA, USA). Helium (99.999%) was used as the carrier gas at a constant flow rate of 1.1 mL min^−1^. The oven temperature program started at 60 °C for 1 min and then increased to 310 °C using controlled ramp rates. Samples were injected in splitless mode (1 μL injection volume) with the injector set to 280 °C. The mass spectrometer operated in electron ionization mode, with both ion source and quadrupole temperatures set to 280 °C. Calibration and quantification relied on a multi-residue standard mixture comprising 217 pesticides (Shanghai Anpel Experimental Technology Co., Ltd., Shanghai, China). Concentrations were calculated according to the Chinese national standard GB 23200.113-2018 adapted for soil matrices. The method limit of quantification was 0.01 mg kg^−1^ dry soil.

### 2.3. 16S rRNA Gene Amplicon Sequencing Analysis

Soil bacterial communities were characterized by high-throughput sequencing of 16S rRNA gene amplicons. We amplified the V3–V4 hypervariable region using primers 338F (5′-ACTCCTACGGGAGGCAGCAG-3′) and 806R (5′-GGACTACHVGGGTWTCTAAT-3′), and performed sequencing on an Illumina MiSeq platform at Majorbio Bio-Pharm Technology Co., Ltd. (Shanghai, China). Raw reads were quality-filtered using fastp v0.23.4 and merged using FLASH v1.2.11. Subsequent downstream sequence processing followed the provider standard Majorbio Cloud (https://cloud.majorbio.com) amplicon workflow. Taxonomic annotation was conducted against SILVA v138 because it remains widely used in 16S rRNA amplicon ecological profiling and is compatible with the provider pipeline and the taxonomy-based functional screening framework used in this study. Taxonomic abundance tables and Bray–Curtis dissimilarity matrices were generated in QIIME v1.9.1. Alpha-diversity indices were calculated in the Majorbio Cloud platform from the genus-level OTU abundance table after sequence quality filtering and taxonomic annotation. Intergroup differences in alpha-diversity indices were evaluated within the same platform, and q-values were used to report FDR-adjusted significance. Sequencing-depth adequacy was assessed using rarefaction curves (Supplementary Figure S1)).

### 2.4. Data Analysis

We compared alpha-diversity metrics among site groups using five biological replicates per group (*n* = 5) by one-way ANOVA followed by Tukey’s HSD post hoc test; when assumptions for parametric testing were violated, we applied the Kruskal–Wallis test. We quantified beta diversity using Bray–Curtis dissimilarities, visualized community patterns by principal coordinate analysis (PCoA), and tested for differences in community structure using ANOSIM and PERMANOVA (Adonis; 999 permutations). We assessed associations between pesticide profiles and bacterial community structure using redundancy analysis (RDA) and distance-based RDA (db-RDA). Constrained ordination analyses were based only on quantified pesticide residue variables. We evaluated concordance between ordinations with Procrustes analysis and tested matrix-level associations using Mantel tests. We considered results statistically significant at *p* < 0.05 unless otherwise stated; for function-level correlation analyses, multiple testing was addressed using Benjamini–Hochberg false discovery rate correction. Taxon-level correlation analysis was used as an exploratory descriptive approach, whereas Benjamini–Hochberg false discovery rate correction was applied to the function-level correlation analysis in Section 3.8.

## 3. Results

### 3.1. Pesticide Residue Gradients

Residual pesticide concentrations showed pronounced spatial heterogeneity and resolved into two clearly separable contamination profiles across the studied soils (mg kg^−1^ dry weight; LOQ = 0.01 mg kg^−1^) (Table 2). Amangeldy (AIN, ANH, AD_10) exhibited a profile consistent with predominantly contemporary inputs, including herbicides and fungicides, together with insecticides from the organophosphate and pyrethroid classes. In contrast, Kyzylkairat (KIN, KNH, KD_10) was dominated by legacy OCPs, particularly dichlorodiphenyltrichloroethane (DDT) transformation products and β-hexachlorocyclohexane (β-HCH), with only limited occurrence of current-use active ingredients.

Within the organochlorine group, DDT and its transformation products were consistently detected only in Kyzylkairat (KIN, KNH; to a lesser extent KD_10), whereas all DDT-related compounds in Amangeldy (AIN, ANH, AD_10) were below the detection limit. Among DDT residues, p,p′-DDE predominated (1.11 mg kg^−1^ in KIN and up to 1.43 mg kg^−1^ in KNH), consistent with the accumulation of aged DDT residues. Reduced metabolites were also substantial: p,p′-DDD occurred at 0.37–0.42 mg kg^−1^ in KIN/KNH, while the parent isomers were present at lower levels (p,p′-DDT: 0.16–0.17 mg kg^−1^; o,p′-DDT: 0.21–0.22 mg kg^−1^). In KD_10, DDT-related signals were markedly lower (p,p′-DDE = 0.038 mg kg^−1^; p,p′-DDD = 0.013 mg kg^−1^; p,p′-DDT = 0.017 mg kg^−1^; o,p′-DDT = 0.013 mg kg^−1^), and o,p′-DDD and o,p′-DDE were not detected. Collectively, the predominance of p,p′-DDE and p,p′-DDD over the parent DDT in Kyzylkairat is consistent with historical inputs and extensive in situ transformation rather than recent application (Table 2).

HCH isomers displayed a spatial pattern opposite to that of DDT. α-HCH and γ-HCH (lindane) were detected exclusively in Amangeldy (α-HCH: 0.019–0.033 mg kg^−1^; γ-HCH: 0.063–0.093 mg kg^−1^), whereas β-HCH was detected only in Kyzylkairat (0.045 mg kg^−1^ in KIN and 0.11 mg kg^−1^ in KNH). This compositional divergence likely reflects differences in use history and isomer persistence, as β-HCH is generally more stable in the environment than the α/γ isomers.

Organophosphate and pyrethroid insecticides were confined to Amangeldy, indicating localized contemporary inputs. Phosalone showed a strong concentration gradient, peaking at AIN (1.93 mg kg^−1^) relative to ANH (0.17 mg kg^−1^) and AD_10 (0.22 mg kg^−1^). Parathion was consistently detected in AIN, ANH, and AD_10 at lower levels (0.08–0.11 mg kg^−1^) and was not detected in Kyzylkairat. Similarly, pyrethroids occurred only in Amangeldy, where fenvalerate ranged from 0.62 to 1.06 mg kg^−1^ (highest at AIN), whereas cypermethrin was detected at 0.033–0.043 mg kg^−1^ in AIN, ANH, and AD_10; both compounds were below detection in Kyzylkairat.

Among the remaining analytes, herbicides and fungicides contributed disproportionately to the total chemical burden in Amangeldy. Simazine occurred at exceptionally high concentrations (20.0–32.3 mg kg^−1^), with a maximum at AIN (32.3 mg kg^−1^), whereas only a trace level was observed in KIN (0.031 mg kg^−1^) and the compound was not detected in KNH or KD_10. Triadimefon showed a closely matching profile (13.9–17.2 mg kg^−1^ in AIN/ANH/AD_10), was present only at trace concentration in KIN (0.021 mg kg^−1^), and was not detected in KNH or KD_10. Triadimenol (0.11–0.25 mg kg^−1^) and biphenyl (0.023–0.073 mg kg^−1^) were also characteristic of Amangeldy. Dicofol was detected at both locations, but concentrations were substantially higher in Amangeldy (0.70–0.83 mg kg^−1^) than in Kyzylkairat (0.071–0.090 mg kg^−1^), and dicofol was not detected in KD_10. Notably, dicofol sulfone, an oxidative transformation product, was detected only in Kyzylkairat (KIN = 0.065 mg kg^−1^; KNH = 0.39 mg kg^−1^; KD_10 = 0.011 mg kg^−1^), which is consistent with more advanced transformation and/or longer residence time in the environment. Prometryn was detected exclusively in Kyzylkairat (KIN = 0.098 mg kg^−1^; KNH = 0.018 mg kg^−1^).

Overall, Amangeldy (AIN, ANH, AD_10) was primarily characterized by comparatively recent agrochemical inputs (high simazine and triadimefon burdens accompanied by organophosphate and pyrethroid insecticides), whereas Kyzylkairat (KIN, KNH, KD_10) retained a signature of historical organochlorine contamination (DDT metabolites and β-HCH) together with transformation products such as dicofol sulfone. This compositional contrast provides a robust chemical basis for subsequent analyses linking contaminants to microbiome structure and for interpreting shifts in the metabolic functional potential of bacterial communities along sharply divergent residue gradients.

### 3.2. Alpha Diversity of Soil Bacterial Communities

**Figure 2:**
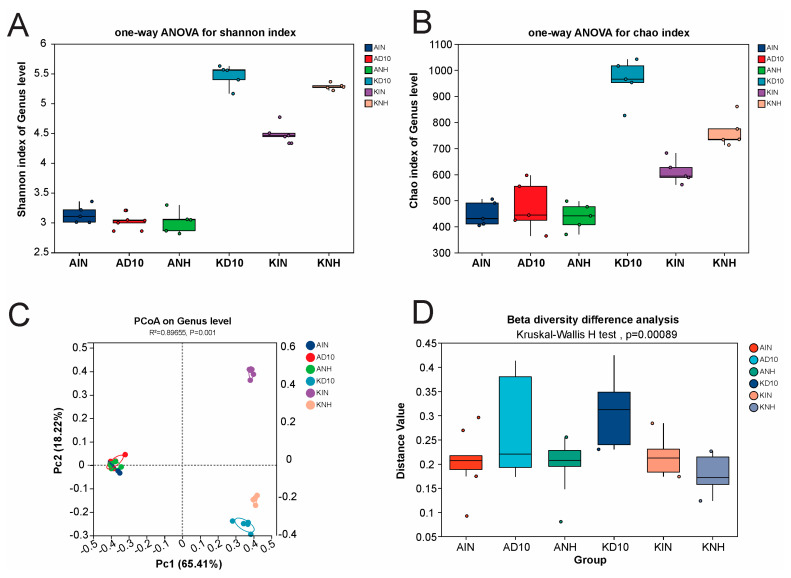
Bacterial alpha and beta diversity among soil samples. Note: (**A**) Shannon diversity index and (**B**) Chao1 richness. (**C**) Principal coordinate analysis (PCoA) based on Bray–Curtis dissimilarities showing separation of samples by treatment; ANOSIM statistics are shown on the plot (R = 0.80, *p* = 0.001). (**D**) Distribution of beta-diversity distance values across treatments; differences among groups were assessed using the Kruskal–Wallis H test (*p* = 0.00089). Abbreviations: AIN—inside the pesticide warehouse area; AD10—10 m from the pesticide warehouse; ANH—neighborhood pesticide warehouse; KD10—10 m from the pesticide warehouse; KIN—neighborhood pesticide warehouse; and KNH—neighborhood pesticide warehouse.

We quantified genus-level alpha diversity using the Shannon index (diversity and evenness) and Chao1 (richness estimator), and compared groups using one-way ANOVA (Figure 2, *n* = 5). Shannon diversity was consistently low in the Amangeldy samples (AIN = 3.1387 ± 0.1490; AD10 = 3.0303 ± 0.1238; ANH = 3.0170 ± 0.1898), whereas Kyzylkairat harbored substantially higher genus-level alpha diversity (KD10 = 5.4634 ± 0.1871; KNH = 5.2856 ± 0.0535; KIN = 4.5032 ± 0.1624). Overall between-group differences were highly significant (*p* = 1.113 × 10^−11^; *p*_adjust = 2.226 × 10^−11^) (Figure 2A).

A concordant pattern emerged for Chao1. Richness estimates remained lower across Amangeldy (AIN = 448.19 ± 46.42; AD10 = 476.84 ± 96.07; ANH = 438.42 ± 51.48) but were markedly higher in Kyzylkairat (KD10 = 960.39 ± 83.48; KNH = 763.18 ± 58.80; KIN = 610.39 ± 46.46), with significant overall group differences (*p* = 1.21 × 10^−6^; *p*_adjust = 1.21 × 10^−6^) (Figure 2B). Collectively, these alpha-diversity metrics indicate a richer (Chao1) and more even (Shannon) bacterial community in Kyzylkairat soils, most prominently in KD10 and KNH.

Rarefaction curves based on the observed species (Sobs) index demonstrated a clear plateau for all samples, indicating that the sequencing depth was fully sufficient to capture the microbial diversity accurately without undersampling bias (Supplementary Figure S1)

### 3.3. Beta Diversity and Community Dissimilarity

The genus-level beta diversity was assessed using PCoA based on Bray–Curtis dissimilarities (Figure 2C). The ordination revealed clear clustering of samples by group, with the first two axes capturing the majority of compositional variation (PC1 = 65.41%, PC2 = 18.22%). Between-group differences in community structure were statistically significant by ANOSIM (R = 0.89655, *p* = 0.001), indicating strong taxonomic differentiation among groups. In addition, within-group dispersion (distance values) differed significantly across groups (Kruskal–Wallis, *p* = 0.00089), consistent with group-specific differences in the degree of community heterogeneity (Figure 2D).

### 3.4. Bacterial Community Structure and Dominant Genera

**Figure 3:**
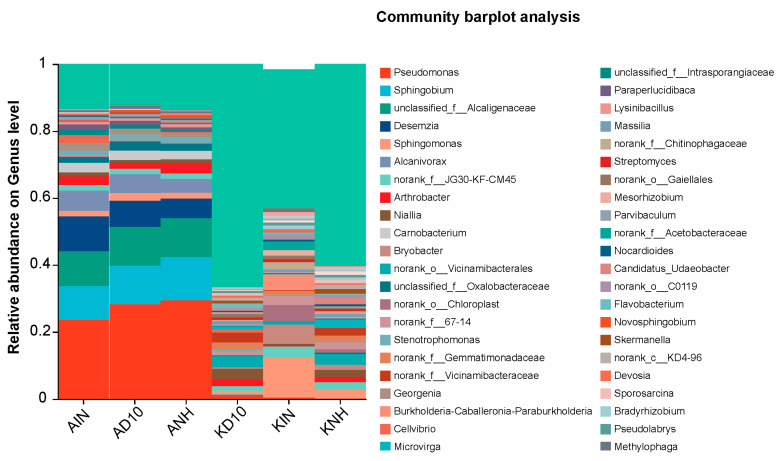
Genus-level composition of soil bacterial communities across sampling groups. *Abbreviations:* AIN—inside the pesticide warehouse area; AD10—10 m from the pesticide warehouse; ANH—neighborhood pesticide warehouse; KD10—10 m from the pesticide warehouse; KIN—neighborhood pesticide warehouse; and KNH—neighborhood pesticide warehouse.

**Figure 4:**
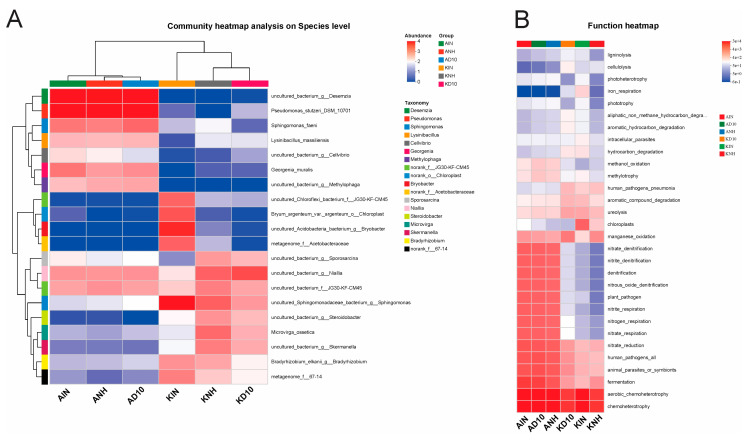
Bacterial community and functional prediction analysis. Note: (**A**) Heatmap of dominant genera across treatments based on group-mean values, with hierarchical clustering using average linkage. Color intensity indicates standardized genus-level signal, and dendrograms depict similarity among soil samples and genera. (**B**) Heatmap of predicted functional groups inferred by FAPROTAX based on 16S rRNA taxonomic annotation. Color intensity indicates the relative abundance of each functional group across soil samples (red, higher; blue, lower). Abbreviations: AIN—inside the pesticide warehouse area; AD10—10 m from the pesticide warehouse; ANH—neighborhood pesticide warehouse; KD10—10 m from the pesticide warehouse; KIN—neighborhood pesticide warehouse; and KNH—neighborhood pesticide warehouse.

Relative abundance profiling at the genus level revealed a pronounced reorganization of dominant taxa across groups (Figure 3). In AIN, AD10, and ANH, the community was consistently characterized by a high relative abundance of *Pseudomonas*, and the co-dominant genera recurrently included *Sphingobium*, *Sphingomonas*, *Desemzia*, *Alcanivorax*, and *Arthrobacter* (Figure 3 and Figure 4A). The heatmap corroborated the consistently higher representation of these genera in AIN/AD10/ANH and their relative depletion in the remaining treatments (Figure 4A).

Conversely, KD10, KIN, and KNH displayed a distinct dominance structure dominated by taxa that are typically associated with soil habitats (Figure 3). These groups showed increased relative abundances of *Bryobacter, Granulicella,* and *Candidatus_Udaeobacter*, as well as lineages assigned to *Vicinamibacterales/Vicinamibacteraceae*, *Gemmatimonadaceae*, and the WD2101 soil group (Figure 4A), indicating a shift toward oligotrophic and soil-adapted assemblages. In addition, KIN exhibited a visually higher representation of the *Burkholderia-Caballeronia-Paraburkholderia* complex, whereas KD10 and KNH showed higher relative abundances of *Bradyrhizobium*, *Microvirga*, *Mesorhizobium*, and *Solirubrobacter* compared with AIN/AD10/ANH (Figure 3). Overall, these patterns indicate a group-specific turnover in dominant genera and a substantive restructuring of community composition across sites.

### 3.5. Prediction of the Functional Potential of Bacterial Communities

We inferred potential functional guilds of the bacterial communities at the genus level using FAPROTAX (Figure 4B). Functional profiles showed strong between-group differentiation, separating Amangeldy (AIN, AD10, ANH) from Kyzylkairat (KD10, KIN, KNH). In AIN/AD10/ANH, predicted functions associated with nitrogen cycling and anaerobic metabolism were elevated, including denitrification, nitrate_denitrification, nitrite_denitrification, nitrate_respiration, nitrate_reduction, nitrogen_respiration, and nitrite_respiration, as well as fermentation. Concurrently, these groups showed higher contributions of chemoheterotrophy and aerobic_chemoheterotrophy, consistent with the dominance of fast-growing, stress-tolerant taxa.

In contrast, KD10/KIN/KNH exhibited reduced relative abundances for most denitrification- and nitrate-respiration-related categories compared with AIN/AD10/ANH, indicating differences in the inferred directionality of biogeochemical processes. However, selected categories linked to transformation of organic substrates including aromatic_compound_degradation, hydrocarbon_degradation, and aromatic_hydrocarbon_degradation were relatively elevated in subsets of the Kyzylkairat groups, which is consistent with a reconfiguration of community functional potential under the site-specific chemical background. Overall, the FAPROTAX results indicate that the divergence between groups extends beyond taxonomic composition to the inferred metabolic functional potential of the bacterial communities (Figure 4B).

### 3.6. Correlation of Pesticide Residues with Dominant Bacterial Taxa

**Figure 5:**
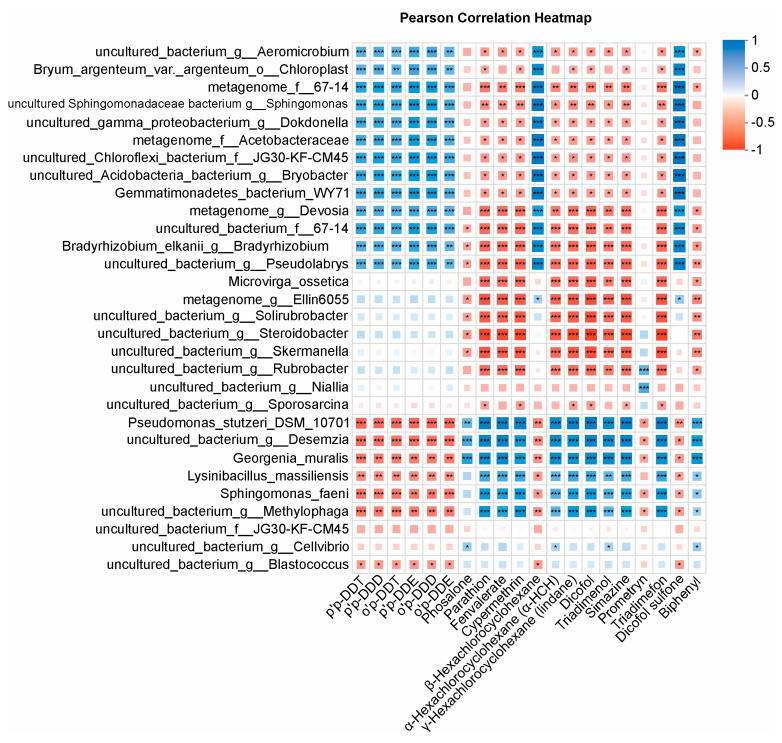
Colors indicate Pearson’s correlation coefficients (r), with blue and red denoting positive and negative associations, respectively. *p*-values are shown as descriptive indicators of pairwise association and should be interpreted cautiously because this analysis was used for exploratory screening. Asterisks indicate statistical significance: * *p* < 0.05, ** *p* < 0.01, and *** *p* < 0.001.

We explored associations between residual pesticide concentrations in soil and the relative abundances of dominant bacterial genera using Pearson’s correlation analysis (Figure 5). Because this analysis was intended as an exploratory descriptive screen of taxon–pesticide covariation, multiple-testing correction was not applied at this level. The heatmap revealed clear directional patterns of association between pesticide variables and dominant taxa, indicating coordinated covariation between pesticide burdens and microbiome composition across samples.

Several taxa showed positive correlations with current-use pesticides, including organophosphates (parathion and phosalone), pyrethroids (cypermethrin and fenvalerate), and triazines (simazine and prometryn), as well as triadimefon, triadimenol, and dicofol. In contrast, DDT-related compounds (p,p′-DDT, p,p′-DDD, p,p′-DDE, and their o,p′ analogs) more often showed negative correlations with these taxa. Conversely, several taxa affiliated with *Acidobacteria/Vicinamibacteraceae* displayed the opposite trend, with positive correlations for DDT/HCH residues and dicofol sulfone and negative correlations for many current-use pesticides. Together, these descriptive patterns indicate marked taxon turnover across contrasting pesticide profiles, while remaining strictly correlative and not implying causality (Figure 5).

### 3.7. The Correlation Between Pesticide Residues and Bacterial Community Structure

**Figure 6:**
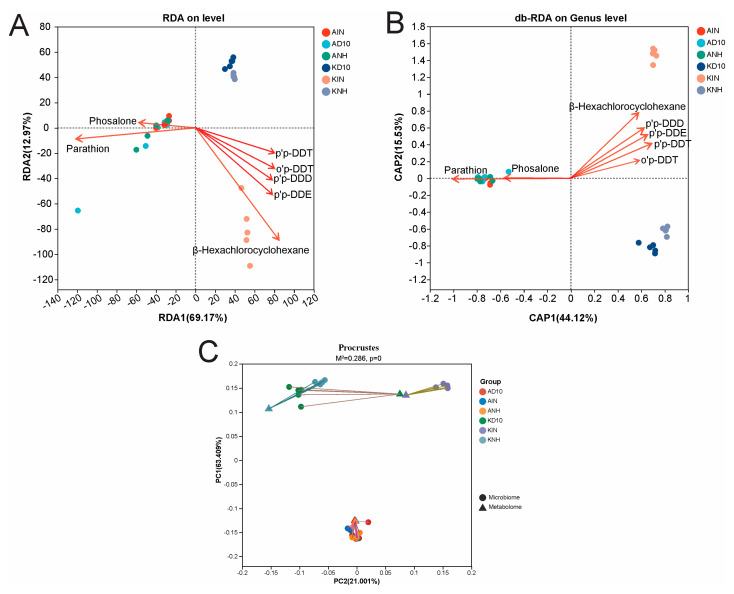
Associations between pesticide residues and soil bacterial community structure. Note: (**A**) RDA ordination based on quantitative pesticide variables; (**B**) db-RDA at the genus level constrained by pesticide residues; (**C**) Procrustes analysis comparing microbiome ordination and pesticide-profile ordination (M^2^ = 0.286, Monte Carlo permutation, *p* < 0.001).

Our analysis revealed the relationship between soil pesticide residue profiles and the taxonomic structure of bacterial communities using constrained ordination and matrix-based approaches (Figure 6A–C). Environmental predictors comprised pesticide concentrations quantified by GC–MS/MS. We tested their explanatory power for microbiome structure using RDA, distance-based RDA (db-RDA; CAP) based on Bray–Curtis dissimilarities at the genus level, and Procrustes analysis to quantify concordance between ordination configurations.

To minimize distortions arising from highly correlated predictors, we screened pesticide variables for multicollinearity using the variance inflation factor (VIF; threshold VIF = 10) (Table 3). After variable selection, four relatively independent predictors were retained for downstream models (all VIF < 10): o,p′-DDT (VIF = 4.53), phosalone (1.84), parathion (3.06), and β-HCH (3.39). This procedure reduces the risk of double-counting tightly co-varying compounds (particularly within the DDT suite) and increases the interpretability and stability of the constrained ordinations.

RDA separated samples sharply along the pesticide gradient (RDA1 = 69.17%, RDA2 = 12.97%; Figure 6A). In ordination space, the vectors for parathion and phosalone were aligned, whereas DDT-associated variables and β-HCH oriented in the opposite direction. This geometry indicates contrasting contamination regimes and shows that the spatial separation of samples is consistent with differences in residue composition (Figure 6A).

db-RDA (CAP) at the genus level using Bray–Curtis distances supported the same conclusion (CAP1 = 44.12%, CAP2 = 15.53%; Figure 6B). The constrained ordination yielded a stable separation of groups, and the primary axis tracked the pesticide gradient parathion-associated profiles loaded toward one pole, whereas DDT markers and β-HCH loaded toward the opposite pole (Figure 6B). Together, these results indicate that variation in pesticide composition is tightly associated with turnover in bacterial community structure.

We further evaluated concordance between pesticide and microbiome configurations using Procrustes analysis (Figure 6C). The analysis revealed strong correspondence between the two ordination spaces (M^2^ = 0.286; permutation *p* < 0.001), indicating that between-sample differences in pesticide residues covaried consistently with between-sample differences in bacterial community composition (Figure 6C).

**Figure 7:**
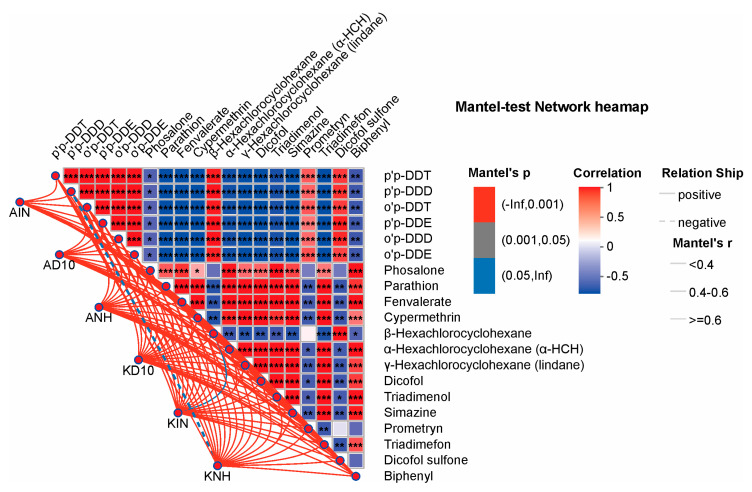
Mantel-test network heatmap summarizing the relationships between pesticide variables and community dissimilarity. Asterisks indicate statistical significance, where *, ** and *** denote *p* < 0.05, *p* < 0.01, and *p* < 0.001, respectively.

In addition, the Mantel-test network heatmap (Figure 7) summarizes the correlation structure among the pesticide variables themselves. DDT-related compounds showed extremely strong positive covariation (e.g., o,p′-DDT vs. p,p′-DDT: r = 0.99968, *p* = 2.75 × 10^−46^; o,p′-DDT vs. p,p′-DDD: r = 0.99876, *p* = 4.82 × 10^−38^), consistent with their joint accumulation and shared variance across samples. Conversely, several current-use pesticides exhibited significant negative associations with DDT metabolites (e.g., fenvalerate vs. p,p′-DDT: r = −0.69119, *p* = 2 × 10^−5^; cypermethrin vs. p,p′-DDE: r = −0.69085, *p* = 2 × 10^−5^; parathion vs. p,p′-DDE: r = −0.69596, *p* = 2 × 10^−5^), indicating the emergence of contrasting “blocks” within the overall residue profile. We also observed strong positive covariation for selected pairs (e.g., dicofol vs. cypermethrin: r = 0.95939, *p* = 6.33 × 10^−17^), whereas other associations were more moderate but remained significant (e.g., phosalone vs. o,p′-DDD: r = −0.38682, *p* = 0.03471). Collectively, these patterns show that pesticide variables cluster rather than vary independently, underscoring why VIF-based multicollinearity control is a critical prerequisite for robust constrained ordination modeling (Figure 7).

### 3.8. Correlation Between Functional Prediction and Microbial Community

**Figure 8:**
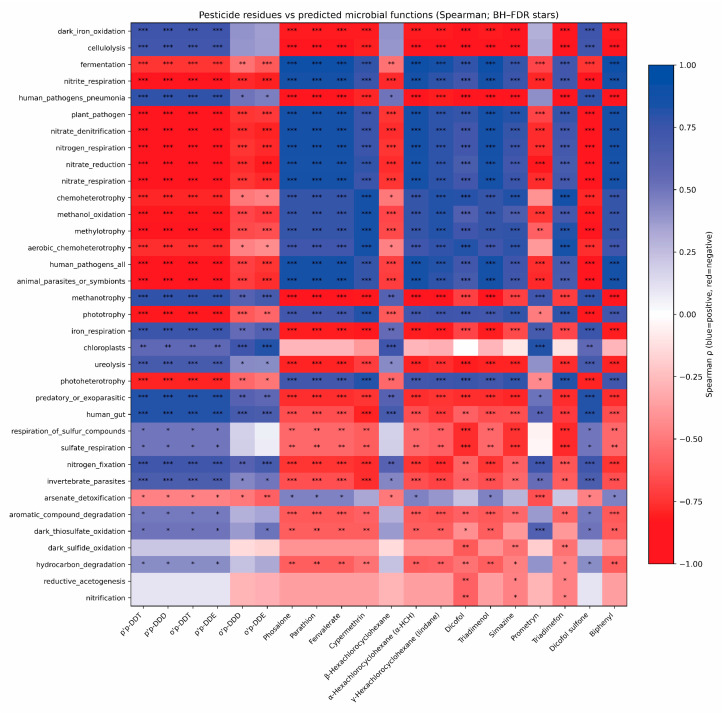
Heatmap of associations between soil pesticide residues and predicted functional categories of the bacterial community (FAPROTAX). Note: Cells show Spearman’s rank correlation coefficients (ρ) for each pesticide–function pair; the color scale indicates the direction and strength of the association (blue, positive; red, negative). Statistical significance was assessed with Benjamini–Hochberg false discovery rate correction (BH–FDR) [21]: * q < 0.05; ** q < 0.01; *** q < 0.001.

The data indicated relationships between residual pesticide concentrations and predicted bacterial functional categories (FAPROTAX) using Spearman’s rank correlation with Benjamini–Hochberg correction for multiple testing (q, BH-FDR) (Figure 8). The heatmap revealed directional and statistically significant associations between multiple pesticides and function-level blocks, indicating coordinated shifts in the community’s inferred metabolic functional potential along the gradient of chemical burden.

The strongest positive associations (Spearman ρ > 0; q < 0.05) involved functions capturing broad heterotrophic metabolism and anaerobic/reductive processes. For instance, fermentation showed consistently high positive correlations with several compounds, including phosalone (ρ = 0.8987, q = 4.44 × 10^−9^), parathion (ρ = 0.8987, q = 4.44 × 10^−9^), and fenvalerate (ρ = 0.8987, q = 4.44 × 10^−9^). Similarly, chemoheterotrophy correlated positively with selected pesticides, such as triadimefon (ρ = 0.8610, q = 5.47 × 10^−8^) and cypermethrin (ρ = 0.8545, q = 8.32 × 10^−8^). Strong positive associations were also evident for methylotrophy-related categories: methanol_oxidation correlated with cypermethrin (ρ = 0.8561, q = 8.11 × 10^−8^), and methylotrophy correlated with cypermethrin (ρ = 0.8545, q = 8.32 × 10^−8^). Collectively, these patterns suggest that variation in pesticide profiles covaries with an increased relative contribution of taxa annotated to heterotrophic organic-substrate utilization and reductive metabolic pathways.

Concurrently, we detected strong negative associations (Spearman ρ < 0; q < 0.05) for several functions linked to polymer degradation and specific redox processes. The most pronounced negative relationships involved cellulolysis with dicofol (ρ = −0.9206, q = 1.80 × 10^−9^), as well as cellulolysis with triadimefon (ρ = −0.9028, q = 4.44 × 10^−9^) and simazine (ρ = −0.8987, q = 4.44 × 10^−9^). A comparable trend was observed for dark_iron_oxidation (e.g., dicofol: ρ = −0.9166, q = 1.80 × 10^−9^; simazine: ρ = −0.8966, q = 4.83 × 10^−9^; triadimefon: ρ = −0.8966, q = 4.83 × 10^−9^). In addition, nitrite_respiration showed a consistent negative association with DDT-related compounds (e.g., p,p′-DDT: ρ = −0.8885, q = 8.92 × 10^−9^). Taken together, this contrast across functional blocks indicates a pesticide-profile-linked reconfiguration of inferred functional potential, while remaining strictly correlative and contingent on taxonomy-based functional annotation (Figure 8).

## 4. Discussion

### 4.1. Chronic Pesticide Stress Bifurcates Microbial Assembly into Distinct r- and K-Selected Trajectories

Our results revealed contrasting community assembly tendencies associated with different pesticide regimes. Soils dominated by modern organophosphates and herbicides, such as simazine and parathion in Amangeldy, were associated with lower alpha diversity (Shannon ≈ 3.03) and a greater relative abundance of copiotrophic taxa such as *Pseudomonas* and *Sphingobium*. In contrast, soils burdened with highly recalcitrant legacy OCPs, such as p,p′-DDE in Kyzylkairat, retained higher diversity (Shannon ≈ 5.46) and were enriched in more oligotrophic and stress-tolerant lineages, including *Acidobacteria*. In this study, the r-/K-framework is used as a literature-guided ecological heuristic to interpret dominant community tendencies, with r-associated assemblages referring to relatively copiotrophic and disturbance-responsive taxa, and K-associated assemblages referring to relatively oligotrophic, persistent, and stress-tolerant taxa. This framework is applied here as an ecological interpretation of the observed taxonomic patterns rather than as a directly measured trait-based classification.

This bifurcated model strongly aligns with Sangwan et al. [14] and Fang et al. [15], as well as with Mahmoudi et al. [22], who demonstrated that long-term legacy OCPs fundamentally alter assembly processes toward K-selected oligotrophic networks. This is further corroborated by Qiu et al. [18] and Sim et al. [23], whose global screenings and large-scale assessments confirmed that different chemical classes impose distinct, predictable taxonomic bottlenecks regardless of soil type. Chen et al. [24] also support this by showing how intensive agricultural management simplifies these co-occurrence networks. However, the broader literature presents stark contradictions regarding chemical filtering. Hoang et al. [25] and Beriot et al. [26] observed no significant alpha-diversity loss in intensive agricultural soils, arguing that root exudates and fertilization mask toxic effects. Furthermore, classic landscape-scale studies, such as those by Delgado-Baquerizo et al. [27], and recent ecotoxicological meta-analyses by Riedo et al. [28] report that specific agricultural management and inherent soil type consistently override the effects of individual pesticide applications, suggesting that chemical residues rarely dictate overall diversity outside of laboratory conditions.

This spectrum of discrepancies is fundamentally explained by the intensity, chronicity, and historical context of the exposure. While acute, low-dose pesticide applications in conventionally managed soils [26] may be buffered by natural soil resilience and transient nutrient inputs, the soils investigated in our study have endured multidecadal, severe chemical accumulation. Under such extreme, uninterrupted toxicity, agricultural buffering mechanisms collapse entirely. The environment permanently shifts into a strongly deterministic state, selecting exclusively for specific life history strategies (r- or K-strategists) that strictly match the biochemical recalcitrance of the prevailing pollutant mixture.

### 4.2. Predicted Functional Reconfiguration of the Microbiome Suggests a Shift Toward Xenobiotic Degradation at the Expense of Baseline Elemental Cycling

FAPROTAX-based functional prediction indicated a marked reconfiguration of inferred community-level functions in the chronically contaminated soils. We observed a higher relative representation of categories related to aromatic compound degradation, chemoheterotrophy, and nitrate reduction, whereas categories such as cellulolysis and dark iron oxidation showed lower relative representation in the more heavily polluted groups. Because these results are based on taxonomy-derived functional inference rather than direct metagenomic or enzymatic measurements, they should be interpreted as predicted functional shifts rather than as direct evidence of pathway activation or suppression.

This pattern is broadly consistent with previous studies reporting that chronic pesticide contamination is associated with enrichment of microbial functions linked to xenobiotic transformation and with restructuring of community-level metabolic potential. Regar et al. [19], Maharana et al. [29], and Zheng et al. [17] similarly showed that long-term pesticide stress can be accompanied by functional reorganization toward degradation-associated processes. However, the broader literature remains mixed. While some studies suggest that contaminated soils may retain or enrich degradation-related potential [19,30], others report that increasing pesticide complexity can impair broader microbial functioning and reduce key ecosystem processes [31,32,33,34]. Additional work has also suggested that the maintenance of catabolic traits may impose energetic trade-offs that constrain other aspects of microbial metabolism under prolonged stress [35,36,37]. One possible explanation for these contrasting findings is the difference between short-term exposure to newly introduced pesticide mixtures and long-term adaptation under chronic contamination. In the latter case, prolonged environmental filtering may favor taxa with greater tolerance to xenobiotic stress and with a higher inferred contribution to degradation-related functions. Nevertheless, this interpretation remains hypothetical and requires direct validation using shotgun metagenomics and process-level measurements.

From an ecosystem perspective, the xenobiotic-associated functional shifts predicted by FAPROTAX may have important implications for soil health. Soils characterized by current-use agrochemical burdens appeared to show a greater relative representation of predicted functions related to nitrogen cycling and fermentation, whereas soils dominated by legacy organochlorine residues were associated with reduced representation of functions linked to baseline organic matter transformation, including cellulolysis. Such shifts may influence nutrient turnover, soil fertility, and ultimately plant productivity, although these links remain inference-based and require direct experimental validation.

### 4.3. The Efficacy of Future Bioremediation Strategies Depends on Ecological Compatibility and Extremotolerance

The distinct community structure observed in the chronically contaminated soils suggests that ecological compatibility may be an important consideration in the design of future bioremediation approaches. In particular, the enrichment of indigenous taxa such as *Microvirga*, *Acidobacteria*, and members of the f_JG30-KF-CM45 clade indicates that these lineages are able to persist under long-term agrochemical stress. Rather than demonstrating remediation efficacy directly, our results identify native community components that may represent promising ecological candidates for future validation in site-adapted restoration frameworks.

This interpretation is broadly consistent with previous studies showing that indigenous pesticide-adapted microorganisms often perform better than non-native inoculants under contaminated field conditions [36]. For example, Alvarez et al. [20] reported enhanced HCH degradation by indigenous *Sphingobium* strains, while Bhatt et al. [37], Megharaj et al. [38], and Fathima et al. [39] emphasized that communities with prior exposure to chronic pesticide stress may retain higher detoxification potential than naive isolates. Srivastava et al. [40] likewise argued that future microbial engineering strategies for sustainable agriculture should increasingly consider field-adapted indigenous consortia.

At the same time, the broader bioaugmentation literature remains highly inconsistent with respect to field performance. Cycoń et al. [41] and Abbas et al. [42] showed that strains performing well under laboratory conditions frequently fail to establish or remain effective in situ. Similarly, Mrozik and Piotrowska-Seget [43] and Gentry et al. [44] highlighted that introduced degraders often face strong abiotic stress, fluctuating resource availability, and competitive exclusion by resident microbial communities, all of which reduce their field-scale persistence and performance.

One plausible explanation for these discrepancies is that microbiome function under chronic pesticide exposure depends not only on catabolic potential, but also on long-term ecological compatibility with the resident soil system. In this context, the patterns observed in our study suggest that indigenous assemblages persisting under severe chemical stress may provide a more realistic ecological starting point for future remediation-oriented research than generic exogenous inoculants. However, our study did not directly test pesticide degradation rates, inoculant establishment, or soil recovery outcomes. Therefore, these findings should be interpreted as identifying candidate native taxa and community patterns for future validation rather than as direct evidence supporting a ready-to-implement bioremediation strategy.

### 4.4. Complex Pesticide Mixtures Are Strongly Associated with Spatial Community Turnover

db-RDA showed clear spatial partitioning of samples along contrasting pesticide gradients, while Procrustes analysis further supported significant concordance between pesticide profiles and bacterial community composition (M^2^ = 0.286, *p* < 0.001).

This pattern is consistent with previous studies suggesting that multi-agrochemical stress can contribute substantially to community turnover. Jiao et al. [45] and Nascimento et al. [46] reported that contrasting pesticide regimes were associated with divergent microbial assembly patterns, while Wang et al. [47] showed that long-term herbicide residues can be strongly linked to shifts in soil multifunctionality and microbiome structure. Karas et al. [48] further highlighted the value of microbial indicators in ecotoxicological assessment frameworks. At the same time, this interpretation should be considered in the context of broader soil ecological theory. Large-scale studies, including those by Delgado-Baquerizo et al. [27] and Fierer and Jackson [49], have shown that basic edaphic properties, especially soil pH and organic carbon, are major determinants of bacterial biogeography. Other studies, such as Némethová et al. [50] and Zhao et al. [51], likewise indicate that inherent soil properties may mediate or buffer the effects of agrochemicals.

Within the present study system, the long-term accumulation of high pesticide burdens may have imposed a sufficiently strong disturbance to generate the observed community separation along residue gradients. However, because baseline edaphic metadata were not available for the present dataset, these findings should be interpreted as evidence of strong association between pesticide profiles and microbial turnover rather than exclusion of other environmental contributors.

### 4.5. Methodological Limitations

While our study provides clear evidence of microbiome restructuring under pesticide stress, several methodological limitations should be acknowledged. First, our observational field design does not permit strict causal inference. Because pristine historical controls and baseline edaphic metadata, including soil pH, total organic carbon (TOC), C:N ratio, and soil moisture, were not available for the present dataset, their contribution to the observed microbial patterns cannot be excluded. Constrained ordination analyses were based only on quantified pesticide residue variables. Nevertheless, the strong covariation between pesticide concentration gradients and bacterial community shifts observed in the multivariate analyses suggests that pesticide residue composition was an important factor associated with microbiome variation within the studied system [52]. Second, our functional interpretation relies on 16S rRNA gene amplicon profiling coupled with predictive FAPROTAX annotation rather than direct metagenomic or enzymatic measurements.

## 5. Conclusions

This study demonstrates that contrasting pesticide residue profiles in the Almaty Region were strongly associated with divergent patterns of soil bacterial community structure and inferred functional potential. Amangeldy soils, characterized by high burdens of current-use agrochemicals, exhibited lower alpha diversity and a higher relative abundance of copiotrophic taxa such as Pseudomonas, whereas Kyzylkairat soils, marked by legacy organochlorine residues, retained higher diversity and were enriched in stress-tolerant oligotrophic lineages such as Acidobacteria. Procrustes analysis supported a significant concordance between pesticide composition and microbiome structure (M^2^ = 0.286, *p* < 0.001). Together, these findings suggest that pesticide residue composition is an important ecological correlate of bacterial community turnover in chronically contaminated semi-arid soils and may provide a basis for future bioindicator development and remediation-oriented studies.

## Figures and Tables

**Table 1:** Description of soil sampling locations and corresponding group codes.

Site	Sample Group Code	Coordinates	Description
Kyzylkairat	KIN	43°17′58.80″ N, 77°11′40.30″ E	Inside the pesticide warehouse area
	KNH	43°17′58.96″ N, 77°11′40.30″ E	Neighborhood pesticide warehouse
	KD10	43°17′58.80″ N, 77°11′40.74″ E	10 m from the pesticide warehouse
Amangeldy	AIN	43°17′55.30″ N, 77°12′27.80″ E	Inside the pesticide warehouse area
	ANH	43°17′55.46″ N, 77°12′27.80″ E	Neighborhood pesticide warehouse
	AD10	43°17′55.30″ N, 77°12′28.24″ E	10 m from the pesticide warehouse

**Table 2:** Concentrations of residual pesticides in the soil samples.

Pesticide	AIN	ANH	AD10	KIN	KNH	KD10
**Organochlorine pesticides: DDTs and metabolites**						
p,p′-DDT	–	–	–	0.16	0.17	0.017
p,p′-DDD	–	–	–	0.37	0.42	0.013
o,p′-DDT	–	–	–	0.21	0.22	0.013
p,p′-DDE	–	–	–	1.11	1.43	0.038
o,p′-DDD	–	–	–	0.25	0.30	–
o,p′-DDE	–	–	–	0.11	0.11	–
**Organochlorine pesticides: HCH isomers**						
β-HCH	–	–	–	0.045	0.11	–
α-HCH	0.033	0.019	0.024	–	–	–
γ-HCH (lindane)	0.087	0.063	0.093	–	–	–
**Organophosphate pesticides**						
Phosalone	1.93	0.17	0.22	–	–	–
Parathion	0.11	0.08	0.10	–	–	–
**Pyrethroids**						
Fenvalerate	1.06	0.62	0.71	–	–	–
Cypermethrin	0.033	0.034	0.043	–	–	–
**Other pesticides**						
Dicofol	0.83	0.70	0.75	0.090	0.071	–
Dicofol sulfone	–	–	–	0.065	0.39	0.011
Simazine	32.3	20.0	25.8	0.031	–	–
Prometryn	–	–	–	0.098	0.018	–
Triadimenol	0.25	0.11	0.13	–	–	–
Triadimefon	16.2	13.9	17.2	0.021	–	–
Biphenyl	0.073	0.023	0.026	–	–	–

Note: Values are expressed as mg kg^−1^ dry soil. “–” indicates concentrations below the method detection limit (0.01 mg kg^−1^).

**Table 3:** VIF (variance inflation factor) values of environmental factors before screening.

Pesticides	VIF Value
p,p′-DDD	98,811.41667
p,p′-DDT	50,270.65548
o,p′-DDT	93,353.96473
p,p′-DDE	18,124.62312
o,p′-DDD	NA
o,p′-DDE	NA
Phosalone	3.55283
Parathion	62.23877
Fenvalerate	NA
Cypermethrin	NA
β-Hexachlorocyclohexane	59.10417
α-Hexachlorocyclohexane (α-HCH)	NA
γ-Hexachlorocyclohexane (lindane)	NA
Dicofol	NA
Triadimenol	NA
Simazine	NA
Prometryn	NA
Triadimefon	NA
Dicofol sulfone	NA
Biphenyl	NA

Note: NA indicates that the VIF value could not be estimated for the corresponding variable.

## Data Availability

The raw 16S rRNA gene sequencing data generated in this study are publicly available in the NCBI BioProject database under accession number PRJNA1453728. Corresponding sequencing records are accessible through the NCBI Sequence Read Archive (SRA).

## Supplementary Materials

The following supporting information can be downloaded at https://www.mdpi.com/article/10.3390/biology15090712/s1. Figure S1: Rarefaction curves based on the observed species (Sobs) index for soil bacterial communities across all sampling sites.

## Abbreviations

The following abbreviations are used in this manuscript:

OCPs	Organochlorine pesticides
DDT	Dichlorodiphenyltrichloroethane
DDE	Dichlorodiphenyldichloroethylene
DDD	Dichlorodiphenyldichloroethane
HCH	Hexachlorocyclohexane
GC-MS/MS	Gas chromatography-tandem mass spectrometry
LOQ	Limit of quantification
rRNA	Ribosomal ribonucleic acid
PCoA	Principal coordinate analysis
RDA	Redundancy analysis
db-RDA	Distance-based redundancy analysis
CAP	Canonical analysis of principal coordinates
ANOSIM	Analysis of similarities
VIF	Variance inflation factor
BH-FDR	Benjamini–Hochberg false discovery rate
