# Supplementary material for: Environmental Filtering of Bacterial Communities Driven by Pesticide Residue Profiles in the Almaty Region, Kazakhstan
Source: Biology (Basel). 2026 Apr 30;15(9):712. doi: 10.3390/biology15090712 (PMC13162571; doi:10.3390/biology15090712)
Supplement: Supplementary file 1 [file biology-15-00712-s001.zip › biology-4262401-supplementary.pdf]

Supplementary materials

Ecological filtration of bacterial communities under the influence of two pesticide residue profiles in the Almaty region, Kazakhstan

Lazzat Asylbekkyzy[1], Bekzhan D. Kossalbayev[2,5], Fiaz Ahmad[4]*, Jingjing Wang[3], Assemgul K. Sadvakasova[1], Meruyert O. Bauenova[1], Altynbek A. Abseyt[5], Dilnaz E. Zaletova[1]*

Figure S1. Rarefaction curves of bacterial OTUs at a 97% similarity level.

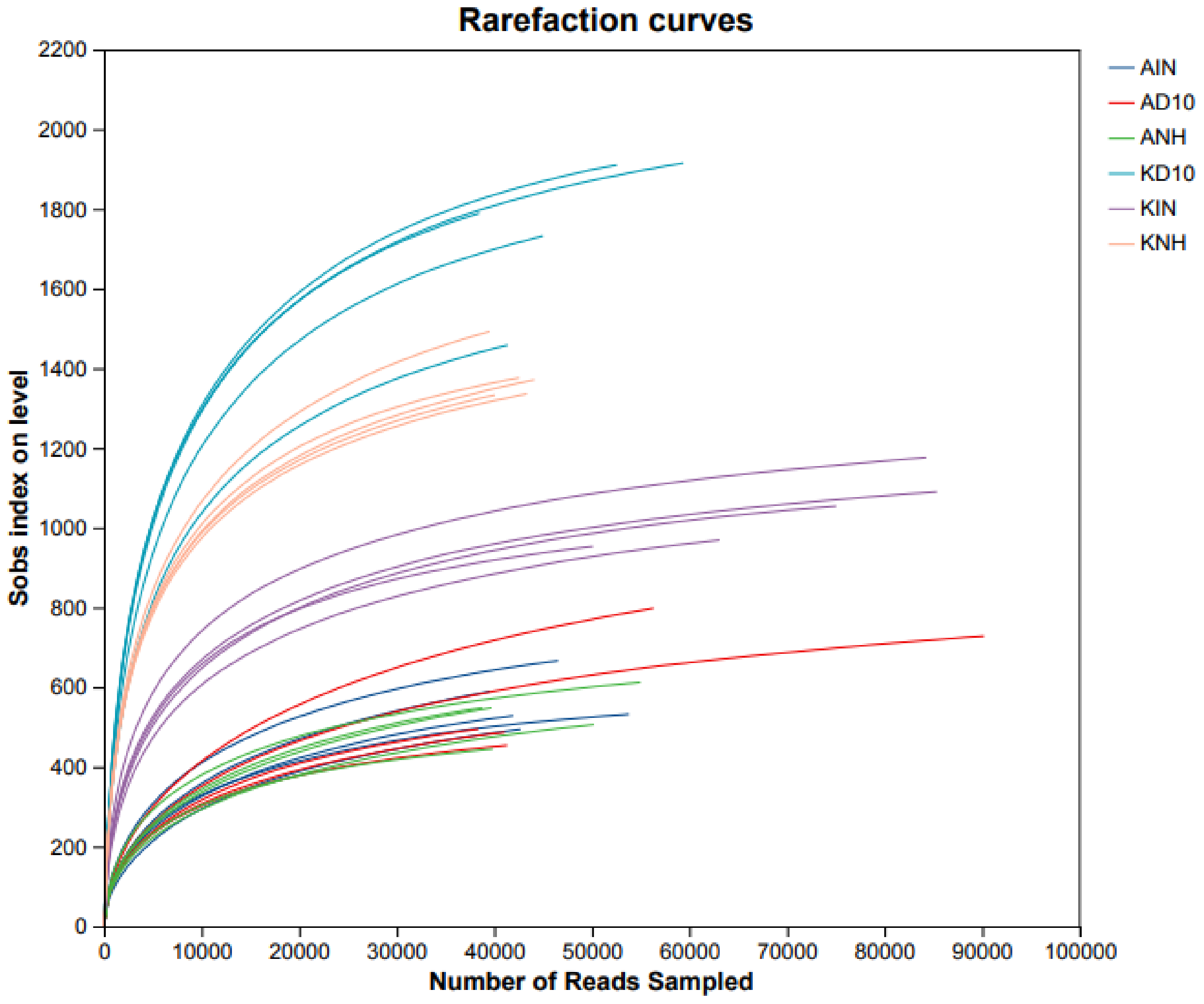

The curves illustrate the relationship between the number of sequences and the number of observed species (Sobs). All samples reached a saturation plateau, indicating sufficient sequencing depth to capture the true taxonomic diversity of the studied soil microbiomes.
